# Effects of heat conduction on the spatial selectivity of infrared stimulation in the cochlea

**DOI:** 10.1186/s12938-015-0017-5

**Published:** 2015-03-14

**Authors:** Kaiyin Zhang, Yue Ma, Yunfei Zhou, Qiuling Wang

**Affiliations:** School of Physics and Electronics, Fuyang Normal College, Fuyang, 236037 Anhui China

**Keywords:** Infrared laser stimulation, Cochlea, Heat conduction, Spatial selectivity, Pulse-trains, Multi-sites stimulation

## Abstract

**Background:**

It has been reported that one of the main mechanisms that induces the activation of the cochlea through infrared laser light is the photothermal effect. The temperature in the spiral ganglion cells increases as a result of photon absorption. However, heat conduction can induce an increase in the temperature within the cochlea and change the spatial selectivity of activation.

**Methods:**

We analyzed the effects of heat conduction on the increase in temperature within the cochlea using a 3D model that simplifies the spiraled cochlea as a rotational symmetric structure . The model is solved using the finite element method.

**Results:**

Taken as an example, the cochlea is stimulated by laser pulses at eight sites in its first turn. The temperature rise in time domain and spatial domain is simulated for different laser pulse energies and repetition rates. The results demonstrate that the temperature in the cochlea increases as the laser pulse energy and repetition rate increase. Additionally, the zone affected by the laser is enlarged because of the heat conduction in the surrounding structures. As a result, more auditory neurons can be stimulated than the expected.

**Conclusions:**

The heat conduction affects the laser spatial selectivity however, by adjusting the stimulation schemes of the laser pulse-trains, such as laser repetition rate and laser power, the laser selectivity can be optimized.

## Background

The success of neuroprosthetic therapy which uses implants in the cochlea for people with hearing loss has resulted in an increasing interest in the artificial stimulation of hearing [1]. It has been suggested that an increasing number of independent perceptual channels can enhance the speech recognition of electronic cochlear implant users in noisy environments [2]. Therefore, further research has been devoted to increasing the number of independent cochlea implant channels through stimulating cochlear spiral ganglion neurons more discretely. In the scheme of electrical stimulation, a number of strategies have been employed to increase the spatial selectivity over a conventional monopolar stimulation paradigm. For instance, multipolar electrode configurations and nerve-penetrating electrodes can increase the spatial selectivity of stimulation. However, these methods are still facing difficulties in improving speech recognition in daily use as the low spatial selectivity is limited by current dispersion [3].

It has been shown that the spatial selectivity of infrared neuron stimulation is similar to or even better than that of electronic implants. A significant advantage of laser stimulation over electrical stimulation is a potential improvement in the spatial selectivity, which can provide more independent channels for simultaneous stimulation of the neural system. The reason is, when compared to electric current, that the laser radiation does not spread significantly in tissues which can confine the optical stimulus to a smaller portion of neurons, allowing more discrete neural stimulation with improved resolution. Although there still exist many research questions to be answered in terms of laser stimulation, the initial success is promising [4,5].

Recently, the use of laser light has been demonstrated by experiments in vivo on gerbils, mice, guinea pigs and cats to stimulate the auditory nerve in acutely and chronically deafened cochleae for a wide range of wavelengths, including 532 nm, 808 nm, 980 nm, 1450 nm, 1850 nm, 1870 nm, and 1940 nm [4-7]. Wenzel et al. demonstrated that visible light (532 nm) with a 10 nanosecond pulse duration can effectively and reliably activate the guinea pig cochlea without any apparent damage [3]. Wang et al. showed that laser stimulation with pulse length at microsecond level can successfully evoke an auditory nervous impulse, similar to acoustic stimulation, by applying a fiber laser system with a wavelength of 980 nm to stimulate guinea pig cochlea [4]. Xia et al. performed laser cochlea stimulation using 808 nm infrared pulses with different pulse duration (100–1,000 μs). Their experiments suggested that the near-infrared with low absorption coefficients can effectively pass through the lymph and stimulate the auditory nerve [5]. The amplitude of CAP evoked by laser stimulation increased monotonically with the increasing laser radiation energy [3-5].

The detailed biophysical mechanism behind laser neural stimulation has been the subject of hot discussions. However, current evidence suggests that one of the possible mechanisms is the photothermal reaction resulting from absorption of laser energy by water in tissue [8-14]. Based on the photothermal mechanism, two secondary processes leading to laser neural excitation have been proposed. One is the changes in membrane capacitance [8], and the other is the direct activation of heat-sensitive ion channels in membrane [9]. Shapiro et al. proposed the changes in membrane capacitance is a result of perturbing the distribution of charged particles in the electrical double layer of the cell membrane by transient laser heating [8]. Albter et al. suggested that TRPV4 channels mediate the infrared laser evoked response in sensory neurons [9]. Using whole-cell patch clamp electrophysiology, Yong and colleagues investigated the laser induced electrical behavior of the gold-nanorod inculbated primary auditory neurons, and obtained a good correlation between the electrical activity of neurons and the temperature rise near the surface of the nanorod-treated auditory neurons [10]. However, applying laser pulses in cochlea could bear a risk of thermal damage to the auditory tissues. In 2013, Matic et al. performed laser implant experiments on cats, in which cochlear function was monitored for more than 6 weeks. Their experiments did not reveal any changes in cochlear function for laser energy ranged from 5–89 μJ/pulse, which confirms the safety of laser stimulation in the cochlea [11]. In order to apply lasers for clinical use, it is important to determine the safe laser parameters to avoid excessive heating.

To the best of our knowledge, there is a lack of direct measurements of the temperature rise in cochlea in vivo during laser stimulation, due to the complicated cochlea structure and the deep location in bulla on the experimental approach. Fortunately, numerical modeling can overcome these experimental limitations to analyze the spatial and temporal temperature rise in cochlea. Thompson et al. developed a numerical model in which the guinea pig cochlea was considered as a two-dimensional three-layered system, which includes perilymph, nerve tissue, and a 10 μm thick bone layer between the nerve and perilymph [15,16]. They studied light absorption in neural tissues by using the Monte Carlo method, and investigated the effects of different stimulation parameters on cochlea heating, such as stimulation rate, stimulation site spacing, fiber core size, light wavelength and pulse duration. Liljemalm et al. presented a model for infrared laser heating of neural tissues and investigated the effects of various parameters, such as laser power, pulse length, frequency of the pulsing and temperature of the surrounding media [14]. Those theoretical models can give detailed information about the spatial and temporal temperature rise in the heated tissues during infrared laser stimulation. However, those models for studying laser-cochlear interaction simplified the coiled cochlea by a 2D structure, and considered the deep part of the spiral ganglion cells as one boundary connecting to a heat reservoir with a constant body temperature. These 2D models work well in studying the heating process for laser stimulation at a single site. However, in a real situation the spiral ganglion of cochlea is located in the periphery of the modiolus. When multiple sites in the spiral ganglion are stimulated by laser pulses, heat can transfer from the laser targets to the central region of the modiolus, and be accumulated there. Heat accumulation in the central region of the modiolus may stimulate unexpected auditory nerves. Therefore, the predication of the laser induced heating may be insufficient with using a 2D model in which the modiolus is set as a heat reservoir with a constant temperature.

In this work, a symmetric 3D model is applied to investigate laser heating in human cochlea. The purpose is to investigate the laser induced temperature rise, and study the heat transfer effects on the laser spatial selectivity.

## Methods

The 3D model for simulating the temperature variations in the cochlea during direct irradiation with infrared laser pulses, is based on the following assumptions.

**Figure 1:**
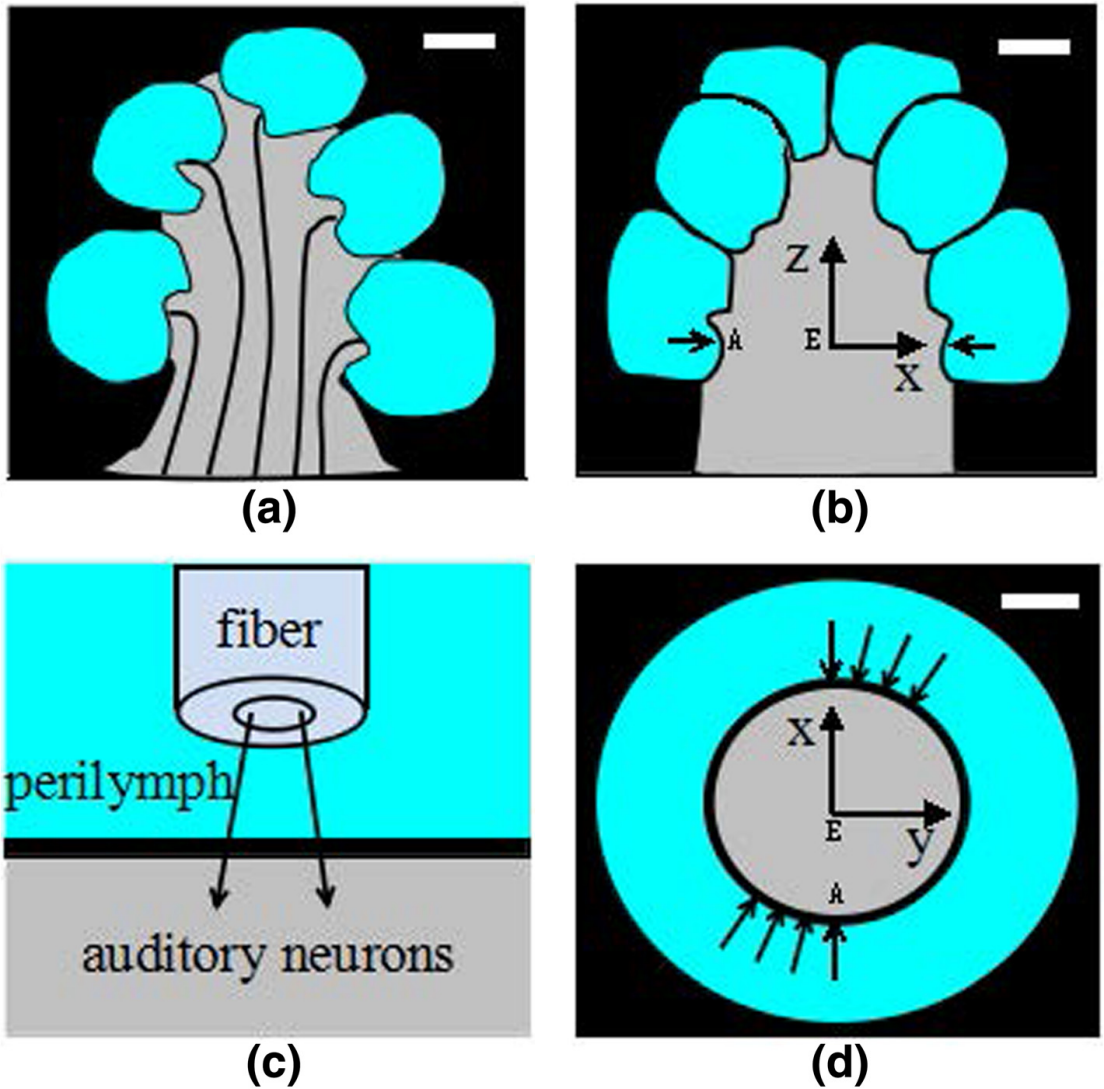
**Diagram of the simplified geometry of the cochlea and the laser stimulations scheme in the 3D modeling. (a)** Illustration of the cross section of a human spiraled cochlea consisting of a spiral tube surrounding the modiolus. **(b)** Cross section of the rotational symmetry model in xz plane utilized in the 3D modeling. **(c)** Geometry of the laser-tissue interaction in the cochlea. **(d)** Illustration of the scheme that eight sites in the first turn of the cochlea that are stimulated by pulse-trains. The white scale bars in **(a),(b)** and **(d)** represent 1 mm, and the characters A and E in **(b)** and **(d)** mark the two sites where the temperature rise vs, time is presented in the following Figure 2.

(1) The spiraled cochlea (Figure 1a) is regarded as a rotational symmetric structure (Figure 1b) for simplification. This offers the opportunity to perform numerical simulations in 3D for multiple site stimulation with a limited additional amount of computational effort. Frijns et al. utilized such a rotational symmetric model to study the spatial selectivity in electric cochlear implant [17]. Their results proved that the rotational symmetric model provides a valuable mean to study the relative influence of various parameters on spatial selectivity. Therefore, a similar scheme is applied in this study.

(2) We assume the human cochlea is stimulated on eight sites in its first turn, as shown in Figure 1b and d. The distance between the laser heating sites and the air tympanic cavity is kept more than 2 mm apart. Therefore, the effects of air convection in the tympanic cavity on laser heating is negligible. The main loss of laser heating is via heat conduction to the skull which is considered a heat reservoir whose temperature is kept at body temperature.

(3) The spiral-shaped cavity of the cochlea has three fluid-filled parts: scala vestibuli, scala tympani and scala media [18]. The center of the cochlea is called the modiolus and has a conical shaped central axis consisting of a spongy bone and the spiral ganglion cells which transfer the auditory information from the hair cells to the brain. To simplify the modeling, we omit the differences in thermal and optical properties of fluids of the three chambers, and use water’s thermal and optical properties for all the fluids within the cochlea (Table 1). Thus, the three chambers of the cochlea are modeled as one chamber, and the spiral cochlear structure is simplified to a rotational symmetric structure, as shown in Figure 1b and c.

**Table 1:** **Thermophysical properties of cochlear tissues**

**Tissue**	**Heat capacity (J/kg/°C)**	**Density (kg/m** ^**3**^ **)**	**Heat conductivity (W/m/°C/)**
Modiolus	3.60 × 10^3^	1.05 × 10^3^	0.51
Perilymph	4.18 × 10^3^	1.00 × 10^3^	0.58
Bone	1.30 × 10^3^	1.90 × 10^3^	0.32
Optical fiber	0.70 × 10^3^	2.60 × 10^3^	1.05

(4) In general, there are three ways of heat transfer in a material: radiation, convection and conduction. For an ideal thermal radiator with a temperature T_1_, the net power radiated per unit area to its surrounding media with a temperature T_2_ is given by *E*_*b*_ = *σ*(*T*_1_^4^ − *T*_2_^4^) in which σ is the Stefan-Boltzmann constant [19]. In the case of laser heating for auditory stimulation, the temperature rise is generally a few degree. By assuming a temperature rise of 2°C, an estimation of the net power radiated can be obtained as being *E*_*b*_ ≈ 13.5*W*/*m*^2^. However, the amount of energy that flows through a unit area per unit time via heat conduction is a function of heat conductivity and temperature gradient, written as *q* = − *k*∇*T* [19]. For a temperature difference of 2°C at a distance of 0.5 mm, the temperature gradient is 4000 K/m. For tissues with an average heat conduction of 0.5 W/m/°C, the heat flux density q is about 2000 *W*/*m*^2^. So thermal radiation is omitted in our model.

Regarding to thermal convection, it can occur mainly in two media in the cochlea. One is the perilymph in the scala tympani and the other is blood in blood vessels and arteries. For a patient who needs a future laser based cochlear implant, the fluid movement in the perilymph can be prohibited technically. So the possible thermal convection in the perilymph is the nature convection due to laser heating induced density difference. However, the onset of nature convection in a closed cavity is decided by the Rayleigh number (Ra) of the cavity. Will nature convection occur only when the Ra is larger than the critical Rayleigh number (R_crit_) of the liquid [20]. In this work, the temperature rise is less than 10 K, and the diameter of the scalar tympani is about 2 mm. Assuming that the perilymp has the same physical properties as water, we found that the Ra of the perilymp in the cochlea is about 113. It is less than the R_crit_ (about 1076) of water [21]. Thus the thermal convection in the perilympy is ignored too.

In laser irradiated tissues, blood flow in blood vessels and arteries has an effect on heat transfer. The diameter of these vessels and arteries is about 10 μm, which is smaller than the laser beam size. Additionally, the velocity of blood flow is about 200–500 μm/s which is slow [22], and thus the forced thermal convection by blood flow is omitted.

In this work, the time-dependent heat conduction model is applied, as given in the following equation [19],

1 $$ c\rho \frac{\partial T}{\partial t}-\nabla \times \left(-k\nabla T\right)=Q $$

where c is the heat capacity at constant pressure (J/(kg °C)), ρ is the density (kg/m^3^), k is the thermal conductivity (W/(m·°C)), and Q is the laser sources (W/m^3^) in the cochlea.

To our knowledge, there is a lack of information detailing physical properties of the cochlea tissues, such as heat conductivity, heat capacity and optical absorption coefficients. However, they can be acquired by analyzing the data of similar structures described in literature [23]. In this study, the laser beam is a few hundred micrometers which is larger than the size of cells such as those spiral ganglion, therefore the effects of individual cells on the physical properties are ignored. Therefore, we take the same approximation method, as illustrated in Figure 1c, for the cochlea as Thompson et al. applied [15]. For example, we took the physical parameters of water for the perilymph in the tympanic channel. The detailed setting of the parameters in our model is listed in Table 1.

**Figure 2:**
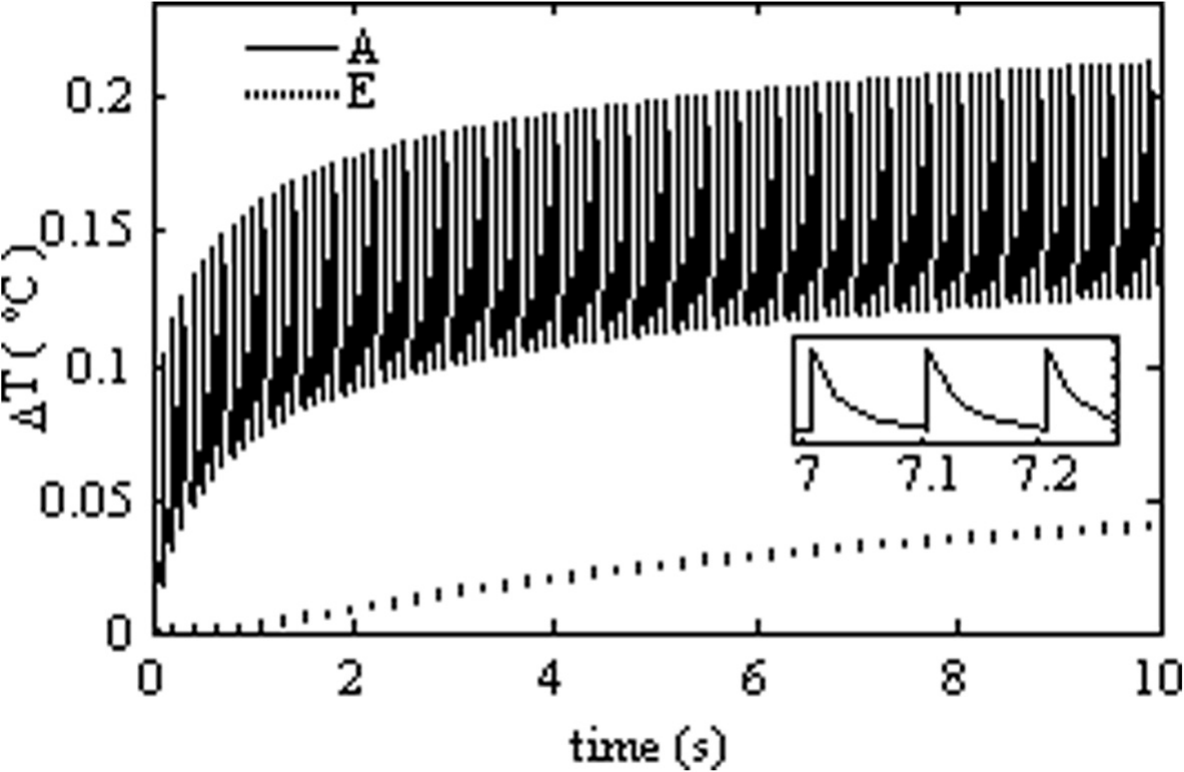
**Temperature change at two typical sites (A and E as marked in Figure** 1 **) in the cochlea (laser pulse energy E = 25 μJ with pulse rates of 10 Hz).** Site A represents the nerve layer 100 μm underneath the osseous spiral lamina, and site E represents the center of the modiolus. The inset shows the temperature variation between three pulses heating. The peak temperature rise at A after 10 seconds is about 0.2°C, while it is less than 0.05°C at E.

For infrared laser stimulation, photons must pass through the perilymph and the osseous spiral lamina, and then reach the spiral ganglion cells (Figure 1c). We choose the laser wavelength 1850 nm as an example, because Izzo et al. reported that nearly one third of the laser energy at this wavelength can reach the spiral ganglion cells [7]. Figure 2b and d illustrate laser stimulation at multi-sites in the human cochlea. The direction of each arrow represents the direction ($\overrightarrow{k}$) of optical fibers in the cochlea. For example, in our calculations we kept the distance between optical fibers and surface of the osseous spiral lamina as 300 μm [24-28]. The laser pulses delivered by the optical fibers to the cochlea are functions of stimulation positions and time, and are written as the following,

2 $$ Q={\displaystyle \sum_{n=1}^8{Q}_0\cdot {e}^{-\frac{\alpha }{k}\left[{\overrightarrow{k}}_n\bullet \left(\overrightarrow{r}-{\overrightarrow{r}}_0\right)\right]}}\cdot {e}^{-\frac{{\left|{\overrightarrow{k}}_n\times \left(\overrightarrow{r}-{\overrightarrow{r}}_n^o\right)\right|}^2}{k^2{\omega}^2}}\cdot {P}_n(t) $$

where n represents the n^th^ laser stimulated site in the cochlea, Q_0_ is the power density at the outlet surface of the optical fiber and can be obtained from the laser beam diameter (ω) and the laser pulse length (τ), α is the light absorption coefficient, ${\overrightarrow{k}}_n$ represents the fiber direction at the n^th^ site, ${\overrightarrow{r}}_n^o$ is the coordinate of the fiber output surface at the n^th^ site, and P(t) is a time dependent dimensionless function representing the laser pulse-trains in time domain. The laser beam is in Gaussian shape and its size can be obtained by taking optical fiber size and light dispersion in tissues into considerations.

## Results and discussion

The 3D model is solved by means of finite element method with COMSOL Script 1.3. The mesh elements are set in a tetrahedron shape with different sizes which are set to be small in the laser irradiated zones and slowly increased for the regions far away from the laser stimulated sites. In total, the 3D model is divided into approximately 50,000 elements, and about 9000 mesh points.

### Temperature rise in time domain

It is important to get an overview of the temperature rise when the cochlea is stimulated at multiple sites by laser pulse-trains. In order to observe the maximal heating effect, we applied a critical stimulation scheme in which all the eight sites (as illustrated by the arrows in Figure 1c) are stimulated by laser pulse-trains with a repetition rate between 1 Hz and 200 Hz. The eight sites are located in the first turn of the human cochlea, and the laser pulse energy and pulse length are kept 12–60 μJ and 100 μs respectively, as these laser parameters have been generally utilized in a number of studies [3-6,24-27].

Figure 2 shows the temperature variations at two representative sites in the human cochlea induced by 10 seconds of laser stimulation with laser pulse energy of 25 μJ and repetition rate of 10 Hz. One site, called A, representing the laser targeted ganglion neurons is localized at about 100 μm underneath the osseous spiral lamina, and the other site, called E, which is not targeted directly by the laser beam is localized at the center of the modiolus. Both sites are marked by the character A and E in Figure 1b and d. The results show that the temperature at both sites increases slowly as more laser pulses are applied. An oscillation of the temperature rise at site A is observed. The site A is located in the laser irradiated region, therefore the tissues at this site get hot directly from laser absorption, which results in an immediate transient temperature raise. After one pulse heating, the temperature decays slowly via heat transfer to the cool regions that are not irradiated by the laser pulses. When the site is irradiated by the following laser pulse, its temperature gets an immediate transient rise and then decays again. Such processes are repeated at the laser repetition rate leading to the oscillation of the temperature change at site A. In contrast, the temperature rise at site E is a result of heat conduction from the laser irradiated zone. Because heat conduction is a slow process compared to the laser repetition rate, there is no fluctuation observed for the temperature rise at site E. In 10 seconds, site A gets a peak temperature rise of about 0.2°C, while the temperature at the center of the modiolus (site E) only increases by 0.04°C. These results are in agreement with the estimation on the temperature rise given by Izzo et al. [27].

### Dependence of the temperature rise on laser repetition rate and pulse energy

In most experiments in vivo, a cochlea was stimulated by laser pulses with a repetition rate between 2 Hz and 250 Hz [24-28]. For a given laser power, the laser repetition rate plays an important role in heating effects within the cochlea.

**Figure 3:**
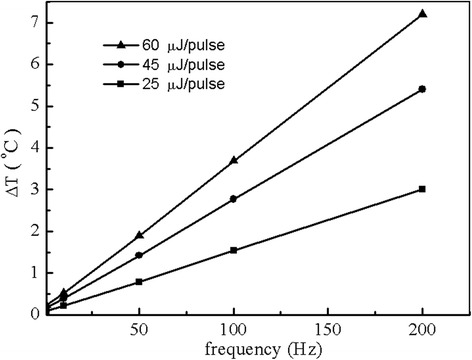
**Repetition rate dependence of the peak temperature rise at ganglion neurons after 10 seconds of stimulation in the cochlea for three given laser pulse energies (E = 25 μJ, 45 μJ and 60 μJ respectively).**

**Figure 4:**
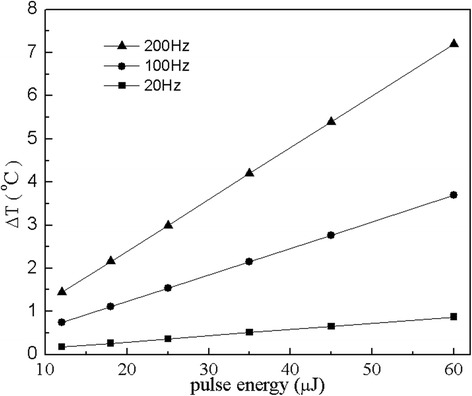
**Pulse energy dependence of the peak temperature rise at ganglion neurons after 10 s of stimulation in the cochlea for three laser repetition rates (f = 20 Hz, 100 Hz and 200 Hz respectively).**

Figure 3 presents the dependence of the temperature rise on the laser repetition rate at site A after 10 seconds of laser stimulation for three given laser pulse energies that are 25 μJ, 45 μJ and 60 μJ respectively. The site A is located approximately 100 μm underneath the osseous spiral lamina representing the laser targeted ganglion cells. When the repetition rate is increased, the total pulse number applied to the cochlea becomes more, which results in a higher temperature rise. Therefore, the temperature rise becomes greater as the repetition rate is increased. In Figure 4, the temperature rise in cochlea vs, laser pulse energy is presented for three different repetition rates which are 20, 100 and 200 Hz respectively, from which a linear relation is found. The linear relation between temperature rise and laser pulse energy is a result of a linear absorption of photons by tissues. The results as given in Figures 3 and 4 show that the temperature rise depends on the laser pulse energy and the laser repetition rate. As the laser pulse energy and repetition rate increase, the temperature rise increases linearly. However, this temperature rise is in a safe range, and could be further reduced if the laser stimulation scheme is improved. For example, we can stimulate the target sites in a determined order instead of the simultaneous stimulation used in this paper. In addition, choosing proper laser pulse energy and laser repetition rate can also influence the temperature rise.

### Distribution of the temperature rise in spatial domain

The spiral ganglion cells in the first turn of the coiled cochlear duct account for high auditory frequency and are located outside the modiolus, while the ganglion cells that account for low frequency in the second and the third turn are located in the internal part of the modiolus. The heat diffusion from the laser irradiated zone to its surrounding tissue may reduce the laser selectivity. Thus, it is interesting to have a quantitative overview on the temperature rise in the spatial domain and assess the effects of the heat diffusion on the laser selectivity.

**Figure 5:**
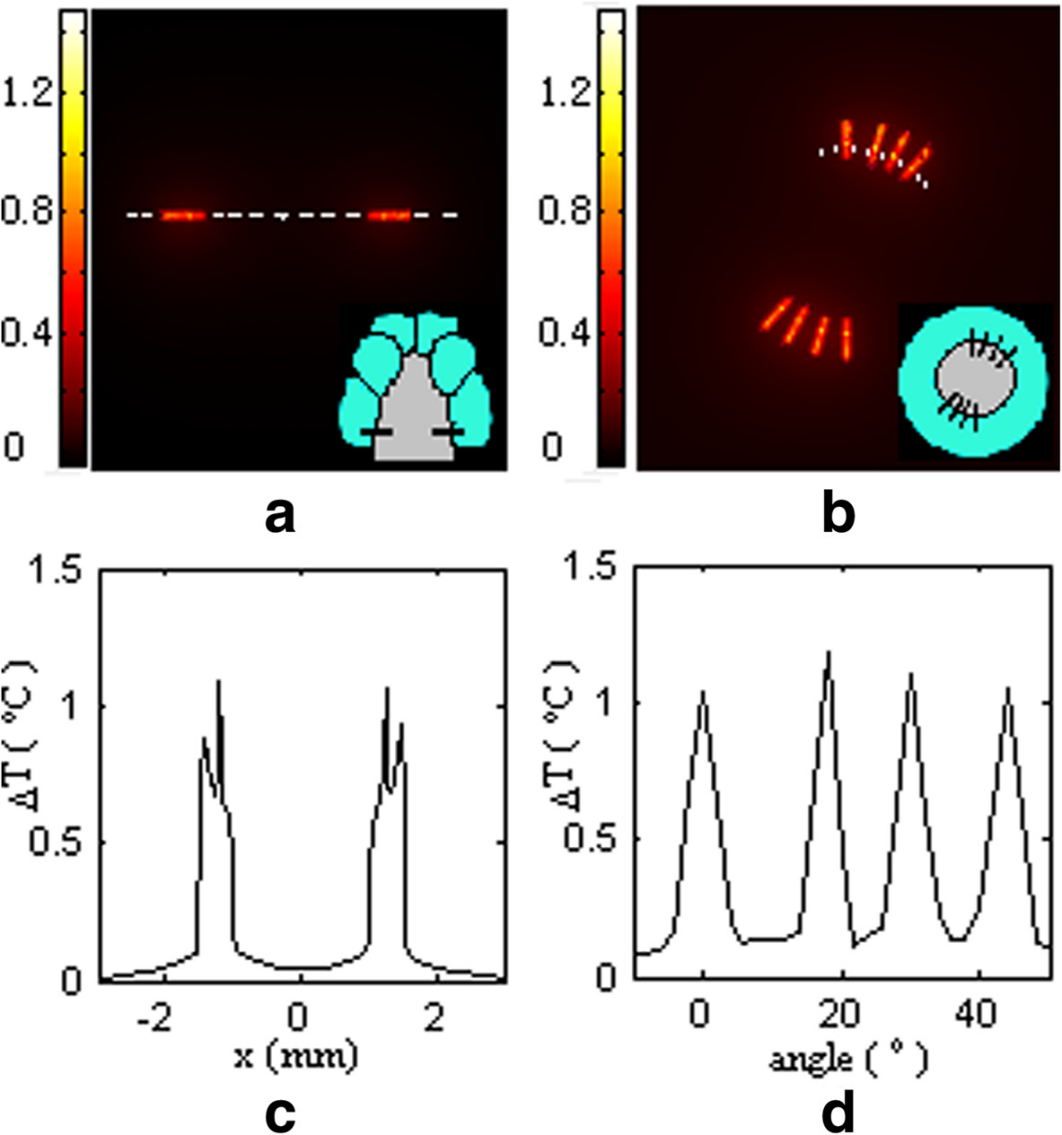
**Distribution of the temperature rise within the cochlea after 10 seconds stimulated by laser pulse-train with laser pulse energy 25 μJ and repetition rate 10 Hz. (a)** Temperature rise in xz plane corresponding to Figure 1b. **(b)** Temperature rise in xy plane corresponding to Figure 1d. **(c)** Temperature rise along the dashed white line in **(a)**. **(d)** Temperature rise along the white curved broken line in **(b)**. The two insets in a and b illustrate the laser stimulation sites in the cochlea.

We calculated the temperature rise in the spatial domain within the cochlea irradiated by the laser pulse-trains that lasts 10 seconds with a repetition rate of 10 Hz and laser pulse energy of 25 μJ. Figure 5a and b present the temperature rise in XZ plane and XY plane corresponding to the Figure 1b and d respectively. In Figure 5a, two stimulation sites are presented, and in Figure 5b all eight stimulation sites are presented. The results show that the absorption of the laser pulses induces heating of the cochlea, and the hot spots are directly located in the zones irradiated by laser beams. But the laser affected zone is larger than the laser illumination due to heat diffusion. Figure 5c presents the profile of the temperature rise along the white dashed line that passes through two laser sites and the modiolus as shown in Figure 5a which corresponds to Figure 1b. From these results we can conclude that: (1) The temperature rise becomes small from the optical fiber to the ganglion neurons in the laser irradiated zones following a decay of laser density; (2) The temperature change is much higher in the osseous spiral lamina because its heat capacity and heat conductivity are small (Table 1); (3) The temperature rise in the modiolus is less than 0.05°C which is about 10 times less than the temperature in the laser targeted ganglion neurons, suggesting that the auditory fibers coming from the second turn of the cochlea can not be stimulated due to heat diffusion.

In Figure 5d, the profile of the temperature rise in the osseous spiral lamina along the white curved broken line (as marked in Figure 5b) is presented. It shows that the temperature rise is about 1°C in the osseous spiral lamina and only 0.1°C in the region outside the laser target region. For a photothermal stimulation, the temperature rise by laser heating is a key parameter for successfully stimulating the auditory neural activation, and the spatial distribution of temperature rise will determine the laser spatial selectivity.

From the results presented in Figure 5c and d, we can see that the heat conduction increased the laser stimulated zone, but the spatial selectivity is not reduced clearly. In this work, eight sites in cochlea are chosen to be stimulated by laser pulses. It indicates that 32 sites can be stimulated by 32 laser beams in the first turn of the cochlea with high spatial selectivity.

**Figure 6:**
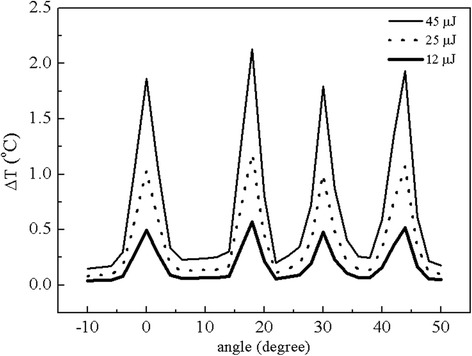
**Temperature rise profile along the white curved broken line in Figure** 6 **b for three different pulse energies. The laser repetition rate is 10 Hz and stimulation time is 10 s.**

Further improvement on the spatial localization of the laser beam can be obtained through adjusting fiber parameters and laser parameters. For example, adjusting laser pulse energy can control the temperature rise and its distribution in the cochlea, and improve the spatial selectivity. Figure 6 presents the temperature rise along the dashed white line in Figure 5b obtained by three different laser pulse energies: 45 μJ, 25 μJ and 12 μJ. We can see that decreasing laser pulse energy can reduce the heat zone and thus increase the spatial selectivity, as shown in Figure 6. Besides, applying lensed and tapered optical fibers (Laser Optics Corp, Israel) can produce a strong laser power density in the target neurons, and induce enough temperature changes in a micron sized region.

## Conclusion

In this paper, the laser induced heating in the human cochlea irradiated by infrared pulse-trains at multiple sites was numerically studied by taking the 3D structure of the cochlea into consideration. In the 3D model, parameters of the laser beam can be adjusted easily to simulate experimental situations, including laser wavelength, laser pulse energy, pulse length, pulse repetition rate, and the distance from optical fiber tip to neurons. Our numerical results show that heat conduction enlarges the laser stimulation zone in the target tissues, and therefore limits on the laser’s spatial selectivity. But the limitation of heat transfer on laser’s spatial selectivity can be controlled by adjusting the stimulation schemes of the laser pulse-trains, such as laser repetition rate and laser power. Further studies may take into consideration the influence of thermal convection via blood flow and other fluids flow on laser heating will improve the model reality.
